# Comparison Between Metabolic Syndrome and the Framingham Risk Score as Predictors of Cardiovascular Diseases Among Kazakhs in Xinjiang

**DOI:** 10.1038/s41598-018-34587-1

**Published:** 2018-11-07

**Authors:** Wenwen Yang, Rulin Ma, Xianghui Zhang, Heng Guo, Jia He, Lei Mao, Lati Mu, Yunhua Hu, Yizhong Yan, Jiaming Liu, Jiaolong Ma, Shugang Li, Yusong Ding, Mei Zhang, Jingyu Zhang, Shuxia Guo

**Affiliations:** Department of Public Health and Key Laboratory of Xinjiang Endemic and Ethnic Diseases of the Ministry of Education, Shihezi University School of Medicine, Shihezi, 832002 China

## Abstract

Metabolic syndrome (MS) and Framingham risk score (FRS) can be used for predicting the risk of developing cardiovascular diseases (CVD). Previous studies that compared FRS and MS have focused on high-income urban areas. This study focused on the comparison between FRS and MS when used in nomadic minorities in mountain areas. Moreover, an applicable tool for predicting the risk of developing CVD was identified. 2,286 participants who were recruited from the far west of China were followed-up for a median of 5.49 years. MS and FRS were compared in terms of their ability in predicting development of CVD using Cox regression and receiver operating characteristic curve. After each component of MS was appraised, its area under the curve (AUC) was 0.647. When age was included, the AUC of MS risk score increased from 0.647 to 0.758 (P < 0.001). After adjusting for age, the AUC of FRS decreased from 0.732 to 0.582 (P < 0.001). The association between CVD and each quintile of MS risk score that included age was more significant than that between FRS and CVD under the same exposed condition. In conclusion, MS risk score that included age may be a better predictor of CVD among Kazakhs.

## Introduction

The prevalence of the risk factors of cardiovascular diseases (CVD) in the Chinese population has increased, causing a higher incidence of CVD^1^. If the underlying cause is not treated, high-risk individuals will develop CVD. Medical treatment is available but is not a permanent solution for CVD. The medical staff will always passively receive and treat patients; however, the harmful effect of CVD toward the patients’ health is challenging to treat. Prevention and control of CVD is now recognized as an urgent public health issue, and early screening and diagnosis are the primary methods of prevention.

Metabolic syndrome (MS), defined as a cluster of cardiovascular risk factors, is a useful tool for the prevention of the rapidly increasing incidence of CVD^2–6^. Numerous studies have shown that MS increases the risk of CVD^7–12^. A meta-analysis has shown that participants with MS were more likely to develop CVD (risk ratio: 2.35; 95% confidence interval [CI]: 2.02–2.73)^13^. Moreover, some studies have found that the prevalence of MS and its related diseases is higher in Kazakhs than in other ethnic groups in Xinjiang^14–16^. Therefore, the understanding of the risk of MS-related CVD in Kazakhs is significantly important. Currently, the American College of Cardiology recommends the use of the Framingham model in predicting the 10-year absolute risk of developing CVD in patients with MS^17^. The Framingham risk score (FRS) is a sex-specific chart that includes age, sex, total cholesterol (TC) level, high density lipoprotein cholesterol (HDL-C) level, smoking status, and systolic blood pressure (SBP)^18,19^. Metabolic syndrome is a cluster of conditions that include increased blood pressure, high blood sugar level, excess fat around the waist, and abnormal cholesterol or triglyceride (TG) levels. However, whether MS is a better predictor of CVD than the FRS remains unclear^20^. Several studies that included western populations have suggested that MS is inferior to the FRS in predicting CVD^3,21–25^. A cohort study involving the Chinese population has found that the MS risk score was a valid tool for predicting CVD, and its predictive ability was as good as the FRS^26^. However, these data cannot be extrapolated in Kazakh populations as their eating habits and living environment are different from other ethnic groups.

Xinjiang is a multiethnic settlement in China, and the Kazakhs are a nomadic nation. They have lived in remote mountain pastures for generations, with limited contact with the outside world. Due to limited resources in public health and poor transportation, significant investigations that analyze local public health needs, including the prevention of CVD, have not been conducted. By contrast, the prevalence of MS in Kazakhs is higher than that of other ethnic groups due to their special ethnicity, living environment, and genetic characteristics^15^. Therefore, evaluation of the prediction models of the risk for CVD in Kazakhs is important in the prevention and treatment of CVD. We conducted a cohort study that compared the predictability of MS and the FRS for CVD to identify the most applicable and simple clinical tool in predicting the development of long-term CVD in the Kazakh population in Xinjiang, China. This study may help identify the appropriate methods to be used in the prevention of chronic diseases, such as CVD, in Kazakhs living in other countries, such as Kazakhstan and Uzbekistan.

## Results

### Baseline Characteristics of the Study Participants

In total, 2,644 individuals participated in the baseline survey (2010–2012) and they were followed-up for more than 5 years on average. A second survey was conducted in 2016 and only 2286 (out of 2,644) subjects were followed-up with a follow-up rate of 86.46%. A total of 278 participants developed CVD during the follow-up period, and the incidence of CVD was 25.24**/**1,000 person-years. Waist circumference (WC), SBP, diastolic blood pressure (DBP), and TC, TG, and fasting plasma glucose (FPG) levels were higher in the MS group (P < 0.001). However, HDL-C levels were lower in the MS group (P < 0.001) (Table 1). Significant differences were observed between the low-, moderate-, and high-risk groups in terms of most covariates (P < 0.001) (Table 2).

**Table 1:** Baseline characteristics of the participants for MS.

Risk factors	MS	Non-MS	*P*
Sex, female/male	330/198	847/630	0.039
Age, years	46.33 ± 12.04	38.71 ± 11.83	<0.001
WC, cm	91.24 ± 10.67	79.40 ± 9.36	<0.001
SBP, mmHg	135.74 ± 18.78	122.97 ± 17.95	<0.001
DBP, mmHg	87.42 ± 12.91	78.71 ± 11.94	<0.001
HDL-C, mmol/L	1.21 ± 0.38	1.41 ± 0.54	<0.001
TC, mmol/L	4.39 ± 1.08	4.00 ± 0.91	<0.001
TG, mmol/L	1.79 ± 1.56	1.06 ± 0.96	<0.001
FPG, mmol/L	5.75 ± 1.66	5.01 ± 0.93	<0.001
Smoking, n(%)	195 (36.93)	434(29.38)	0.001
Drinking, n(%)	80 (15.15)	147(9.95)	0.001
Family history of hypertension, n(%)	206 (39.02)	501(33.92)	0.035
Family history of diabetes, n(%)	9 (1.70)	9(0.61)	0.022
Family history of CVD, n(%)	56 (10.61)	113(7.65)	0.036

Abbreviations: WC, waist circumference; SBP, systolic blood pressure; DBP, diastolic blood pressure; HDL-C, high-density lipoprotein cholesterol; TC, total cholesterol; TG, triglyceride; FPG, fasting plasma glucose; FRS, Framingham Risk Score; CVD, cardiovascular diseases.

**Table 2:** Baseline characteristics of the participants for the FRS.

Risk factors	FRS < 10% Low risk	FRS10–20% Moderate risk	FRS > 20% High risk	*P*
Sex,female/male	1165/642	12/160	0/26	<0.001
Age,years	38.73 ± 10.86	58.14 ± 10.11	63.15 ± 9.65	<0.001
WC,cm	81.75 ± 10.72	89.28 ± 10.82	91.15 ± 14.30	<0.001
SBP,mmHg	124.22 ± 17.23	143.90 ± 22.47	157.19 ± 25.89	<0.001
DBP,mmHg	79.80 ± 11.99	91.27 ± 14.09	96.54 ± 16.98	<0.001
HDL-C,mmol/L	1.36 ± 0.52	1.38 ± 0.40	0.97 ± 0.21	<0.001
TC,mmol/L	4.03 ± 0.94	4.78 ± 0.99	4.46 ± 1.22	<0.001
TG,mmol/L	1.21 ± 1.15	1.45 ± 1.19	2.50 ± 2.79	<0.001
FPG,mmol/L	5.14 ± 1.06	5.83 ± 2.16	5.44 ± 1.19	<0.001
Smoking rate,%	478 (26.45)	130 (75.58)	21 (80.77)	<0.001
Drinking rate,%	163 (9.02)	59 (34.30)	5 (19.23)	<0.001
Family history of hypertension,%	645 (35.69)	57 (33.14)	5 (19.23)	0.181
Family history of diabetes,%	15 (0.83)	2 (1.16)	1 (3.85)	0.445
Family history of CVD,%	150 (8.30)	17 (9.88)	2 (7.69)	0.776

Abbreviations, see Table 1.

### Association between the Components of MS and CVD

The presence of MS was associated with a higher risk of CVD. The risk of CVD increased significantly with increasing number of MS components, and this trend persisted even after adjusting for sex, drinking status, and family history of hypertension, diabetes, and CVD. After adjusting for the above-mentioned risk factors, the participants with ≥3 MS components were more than three times at higher risk of developing CVD than those who without any components (Table 3).

**Table 3:** Association between the components of MS and CVD.

Risk factors	Hazard ratio(HR)	Adjusted Hazard ratio(aHR)
MS*	2.22 (1.75, 2.83)	2.21 (1.73, 2.81)
WC (male ≥ 90 cm, female ≥ 80 cm)	2.28 (1.78, 2.91)	2.21 (1.72, 2.84)
TG (≥1.7 mmol/l)	1.54 (1.14, 2.08)	1.56 (1.15, 2.11)
HDL-C (male<1.04, female < 1.3 mmol/l)	1.24 (0.97, 1.59)	1.18 (0.92, 1.52)
BP (SBP ≥ 130, DBP ≥ 85 mmHg)	2.07 (1.62, 2.64)	2.14 (1.67, 2.74)
PFG (≥5.6 mmol/l)	1.32 (1.03, 1.69)	1.34 (1.04, 1.72)
0 (0 component is abnormal)	1.00	1.00
1 (1 component is abnormal)	1.82 (1.07, 3.12)	1.78 (1.04, 3.05)
2 (2 component is abnormal)	2.86 (1.70, 4.82)	2.73 (1.61, 4.60)
3 (3 component is abnormal)	3.58 (2.09, 6.13)	3.47 (2.02, 5.98)
≥4 (≥4 component is abnormal)	6.41 (3.71, 11.06)	6.13 (3.54,10.60)

HR, the single-factor Cox proportional hazards model; aHR, adjusted for sex, drinking status, and family history of hypertension, diabetes, and CVD.

*NECP-R ATPIII, Revised National Cholesterol Education Program Adult Treatment Panel III.

### Comparison of the Predictive Ability of MS and the FRS

**Figure 1:**
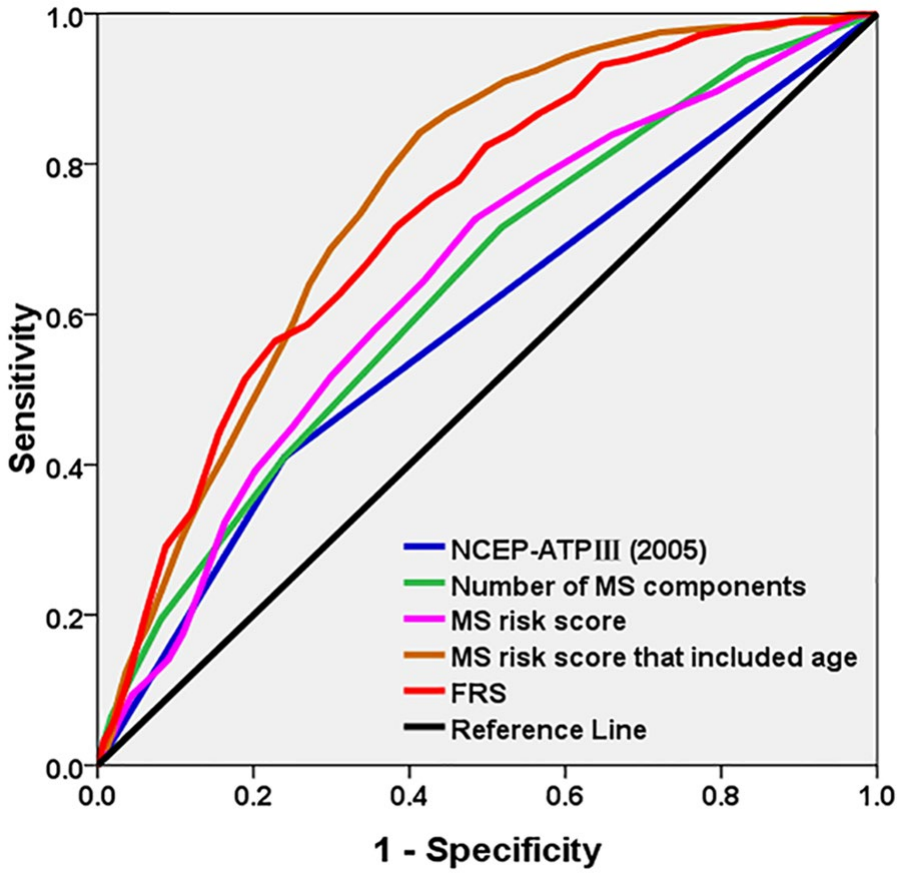
Receiver operating characteristic curves for MS (ATP-III), number of MS components, MS risk score that included age, and the FRS for the prediction of CVD.

MS is a binary variable, and the area under the curve (AUC) of MS in predicting CVD as analyzed using the receiver operating characteristic (ROC) curve was 0.585. Referring to the FRS, we assigned a value to each component of MS to establish the MS scoring system. The total score is equal to the sum of the values of each component of MS, and the risk of CVD was predicted based on the total score. The AUC of the MS risk score in predicting CVD as analyzed using the ROC curve was 0.647. However, the AUC of the MS risk score was still lower than that of the FRS (Table 4, Fig. 1). Furthermore, mutually adjusting the risk factors of MS and the FRS, for a given specificity, after independently adding TC level and smoking status in the FRS to MS risk score or after independently removing WC, TG level, and FPG level from the MS risk score. No significant change was observed in the sensitivity and AUC (P > 0.05). However, when age was included, the sensitivity of the MS risk score increased from 78.1% to 91.0%, and the AUC increased from 0.647 to 0.758 (P < 0.001) (Supplemental Table S3). Meanwhile, WC, TG level, and FPG level that were independently included in MS were added to the FRS, and TC and smoking status were independently removed from the FRS. The sensitivity and AUC did not change (P > 0.05). However, after removing age from the FRS, results showed that the sensitivity and AUC decreased from 84.2% to 66.9% and from 0.732 to 0.582, respectively (P < 0.001) (Supplemental Table S3). In addition, each component of MS was appraised individually and was analyzed using the ROC curve. We found that HDL-C level had the least predictive ability, whereas BP and WC had the best predictive ability (Supplemental Table S4).

**Table 4:** Comparison between the number of MS components, MS risk score, and the predictive ability of the FRS for CVD.

Items	AUC	95%CI	*P*
NCEP-ATPIII (2005)	0.585	(0.548, 0.623)	<0.001
Number of MS components	0.636	(0.601, 0.671)	<0.001
MS risk score	0.647	(0.613, 0.681)	<0.001
MS risk score that included age	0.758	(0.732, 0.784)	<0.001
FRS	0.732	(0.702, 0.762)	<0.001

### MS and FRS and Measures of Probability (Percentage) for the Occurrence of CVD

After adjusting for sex, drinking status, and family history of hypertension, diabetes, and CVD, the hazard ratio (HR) for MS components and MS risk score was lower than that for the FRS (1.78, 2.73, 3.47, and 6.13 vs 1.05, 2.20, 3.01, and 4.17 vs 3.69, 6.36, 8.47, and 16.99), and the HR for each quintile of the MS risk score that included age was higher than that for the FRS(3.51, 9.76, 19.62, and 25.07 vs 3.69, 6.36, 8.47, and 16.99). Furthermore, the participants in the fourth quintile of the MS risk score that included age had a higher probability of developing CVD than those in the top quintile of the FRS (HR: 19.62 vs 16.99) (Table 5).

**Table 5:** Metabolic syndrome and the Framingham risk score and measures of probability (percentage) for the occurrence of CVD.

Quintile	FRS			MS risk score that included age			MS risk score			items	Number of MS components		
	CVD(n)	Incidence of CVD (/1,000 person years)	HR(95% CI)	CVD(n)	Incidence of CVD (/1,000 person years)	HR(95% CI)	CVD(n)	Incidence of CVD (/1,000 person years)	HR(95% CI)		CVD(n)	Incidence of CVD (/1,000 person years)	aHR(95% CI)
Q1	8	3.66	1.00	5	2.29	1.00	29	13.26	1.00	0	17	9.75	1.00
Q2	36	14.14	3.69 (1.71,7.94)	20	7.86	3.51 (1.31,9.37)	32	12.57	1.05 (0.63,1.74)	1	62	17.80	1.78 (1.04,3.05)
Q3	48	23.31	6.36 (3.01,13.46)	49	23.80	9.76 (3.88,24.57)	50	24.28	2.20 (1.37,3.52)	2	85	27.44	2.73 (1.61,4.60)
Q4	63	29.15	8.47 (4.05,17.69)	91	42.10	19.62 (7.96,48.36)	72	33.31	3.01 (1.92,4.74)	3	60	34.00	3.47 (2.02,5.98)
Q5	123	59.67	16.99 (8.30,34.78)	113	54.82	25.07 (10.18,61.75)	95	46.09	4.17 (2.67,6.50)	≥4	54	58.39	6.13 (3.54,10.60)
FRS 10-year risk,%													
≤10	218	21.85	1.00	211	21.15	1.00	237	23.75	1.00	≤1	79	15.11	1.00
11 ~ 20	54	58.21	4.48 (3.11,6.45)	60	64.68	3.44(2.58,4.59)	33	35.57	1.48 (1.02,2.14)	2	85	27.44	1.77 (1.30,2.42)
>20	6	54.98	5.25 (2.24,12.31)	7	64.14	3.04 (1.43,6.48)	8	73.30	2.61 (1.28,5.32)	≥3	114	42.38	2.79 (2.05,3.79)

Note: adjusted for sex, drinking status, and family history of hypertension, diabetes, CVD.

## Discussion

This cohort study included the Kazakh population; the incidence of CVD in Kazakhs was 25.24**/**1,000 person-years, which was higher than the national average in China^26–28^. This difference may be due to the unique eating habits of Kazakhs. That is, they usually consume pasta, beef and mutton, dairy products, and foods high in fat. In addition, the prevalence of hypertriglyceridemia is low in this group. Our investigation showed that participants with MS were more likely to develop CVD than those without. This finding was also reported in previous studies^7,29,30^. Similarly, a meta-analysis by Galassi *et al*.^31^ that examined the association between MS and the risk of CVD has suggested that participants with MS were at high risk of developing CVD.

The present study found that the risk of developing CVD increased significantly with increasing number of MS risk components, and this trend persisted even after adjusting for sex, drinking status, and family history of hypertension, diabetes, and CVD, and the participants with ≥3 MS components were three times at higher risk of developing CVD than those without any components. This result was consistent with the results obtained by Liu *et al*.^32^ who have reported the relationship between MS and CVD in individuals in 11 provinces of China. In recent years, studies conducted in China and other countries had similar conclusions^33,34^. Therefore, consideration of the number of risk components may be more informative than the MS binary classification when determining risk in clinical practice.

Several studies have been conducted to assess the relative advantages of MS and the FRS for the prediction of the risk for CVD. However, the results were inconsistent^19,22–24^. Whether one predicting tool is superior to the other in assessing cardiovascular risk among patients is yet to be determined^20^. However, both MS and the FRS can be effectively used for predicting the long-term risk of cardiovascular events, bearing in mind some of the potential limitations of each tool. To further explore the development of CVD as predicted using MS and the FRS, we conducted this study by making MS components continuous. Referring to the FRS, we assigned a value to each component of MS to establish the MS scoring system. The total score is equal to the sum of the values of each MS component, and the risk of CVD was predicted based on the total score. The AUC of the MS risk score for predicting CVD as analyzed using the ROC curve was 0.647. MS is a binary variable, and the AUC of MS in predicting CVD as analyzed using the ROC curve was 0.585. Therefore, assigning a value to each component of MS was used to establish the MS scoring system compared to MS binary classification, and its ability to predict CVD improved. However, the predictive power of the MS risk score is still lower than that of the FRS (AUC: 0.647 vs 0.732, P < 0.001). Furthermore, the risk factor of MS and the FRS was mutually adjusted in a given specificity^26^ and TC and smoking status in the FRS were independently added to the MS risk score. Moreover, WC, TG level, and FPG level were independently removed from the MS risk score. No significant change was observed in terms of sensitivity and AUC (P > 0.05). By contrast, WC, TG level, and FPG level that were included in the MS risk score were independently added to the FRS, or TC and smoking status were independently removed from the FRS. The sensitivity and AUC did not change (P > 0.05). These indicated that the difference between the FRS and MS in predicting CVD is not due to differences in TC level, smoking status, WC, TG level, and FPG level that were included in the two standards.

Further analysis found that the MS risk score significantly increased the sensitivity and AUC when age was included. That is, the sensitivity and AUC increased from 78.1% to 91.0% and from 0.647 to 0.758, respectively (P < 0.001). The predictive ability of the MS risk score that included age was superior to that of FRS (AUC: 0.758 vs 0.732; sensitivity: 91.0% vs 84.2%; P < 0.05), which indicates that age plays an important role in predicting CVD. By contrast, after removing age from the FRS, its sensitivity and AUC decreased from 84.2% to 66.9% and from 0.732 to 0.582, respectively (P < 0.001). This result showed that some previous studies^21,22^ have considered the relatively lower predictability of CVD for MS compared to the FRS, and this could be explained by the fact that age was not included in the criteria for MS. Therefore, age does play an important role in predicting the development of CVD using either MS or the FRS. YU *et al*.^26^ have shown that age was the best index for predicting CVD in the Jiangsu population in China, which is consistent with the results of the present study. In this study, the AUC and sensitivity of the FRS in predicting CVD were lower than those obtained by Yu *et al*. (AUC: 0.73 vs 0.78; sensitivity: 84.2% vs 87.8)^26^. The possible reason is that the average age of the Jiangsu populations was 50.3 years, whereas the average age of the Kazakh populations was 40.7 years. The average age between the two groups was significantly different, which may further show that the ability to predict the risk of CVD also increased with increasing age. Because the prevalence of MS is high among Kazakhs and MS is a cluster of conditions that include increased BP, high blood sugar level, excess fat around the waist, and abnormal cholesterol or TG levels, the MS risk score that included age may be a better predictor of CVD, and it may be more accurate in reflecting the risk of CVD among Kazakhs in Xinjiang. The MS risk score that included age can identify individuals who are at high risk of developing CVD, thus preventing CVD at an early stage and reducing the burden of CVD. Then, each component of MS was appraised individually and analyzed using the ROC curve. We found that HDL-C level had the least predictive ability, whereas BP and WC had the best predictive ability among Kazakhs.

A multivariate Cox proportional hazards analysis showed that the MS risk score and number of MS components for HR were less than those of the corresponding FRS. The HR values of the participants in each quintile of the MS risk score that included age were higher than those of the corresponding FRS groups, and this result showed that the MS risk score that included age is more associated with CVD than the FRS, which was consistent with the conclusion of a report on the Han Chinese population^26^. In addition, the participants in the fourth quintile of the MS risk score that included age showed a higher probability of developing CVD than those in the top quintile of the FRS. The possible reason for this phenomenon is the high prevalence of MS and abdominal obesity among Kazakhs in Xinjiang. Therefore, the MS risk score that included age may be a better tool than other predicting tools for the Kazakh population with a higher prevalence of MS. Early prediction of CVD in a high-risk population and dietary intervention play an important role in decreasing the incidence of CVD and improving the quality of life.

## Limitation

This study focused on low-income rural areas and nomadic minorities residing in the far west of China. Thus, our findings may not be generalizable to other populations. However, due to similarities in religion, culture, lifestyle, diet, and genetic background in these ethnic groups, our findings may provide some important insights about issues related to the prediction of CVD occurrence in rural Kazakh populations living in other countries, such as Kazakhstan and Uzbekistan. In addition, our findings may have important implications in preventing public health issues for medically underserved Muslim Kazakh minorities. By contrast, molecular studies have shown that the Kazakh populations reside at the borders of countries where Caucasians and Asians are mixed, and there were few similar well-designed prospective studies that were conducted in Asia, particularly those including Muslim minorities. Thus, our findings may be applicable to Caucasians and Asians.

## Conclusions

The predictive ability of the MS risk score that included age was superior to that of the FRS. Moreover, the MS risk score that included age may be a better predictor of CVD, and it may be more accurate in predicting CVD in Kazakhs in Xinjiang.

## Method

### Study Population

First, we chose some representative prefectures (six villages in Nalati Township, Xinyuan County, Yili) according to the geographical distributions of the minority populations in Xinjiang, a province in the northwest of China. Briefly, the baseline study was conducted from 2010 to 2012 in Xinjiang province. In total, 2,644 individuals participated in the baseline survey (2010–2012), and they were followed-up for more than 5 years on average. A second survey was conducted in 2016, and only 2286 out of 2,644 participants were followed-up with a follow-up rate of 86.46%. The median follow-up period was 5.49 person-years (in total 11014.92 person-years). A total of 281 individuals who developed CVD (coronary heart disease [CHD], stroke, and hypertension) at baseline survey were excluded from the analysis. Overall, only 2,005 individuals were included in the cohort analysis. The average age of the participants was 40.72 ± 12.34 years. A total of 278 individuals developed CVD during the follow-up period. The cardiovascular survival time was defined as the duration of follow-up in years from 2010 to the first occurrence of the event or end of follow-up, whichever came first.

### Epidemiological Survey and Biochemical Detection

Data of the participants were collected using a self-administered questionnaire during a face-to-face interview. The questionnaire contained questions regarding the participant’s personal profile, drinking and smoking status, physical exercise, details of existing disease, and family history of diseases. WC, SBP, and DBP were measured by trained field workers in accordance with standardized methods^35^. Blood tests were performed, and TG, TC, HDL-C, and FPG levels were assessed. All blood samples were analyzed using an automatic biochemical analyzer (Olympus AU 2700; Olympus Diagnostics, Hamburg, Germany). Each participant signed an informed consent form. All the described methods were performed in accordance with the approved guidelines and regulations. This study was approved by the Institutional Ethics Review Board (IERB) of the First Affiliated Hospital of Shihezi University School of Medicine (IERB no.: SHZ2010LL01).

### Diagnostic Criteria for CVD

The participants who have one of the following conditions were diagnosed with CVD: the first-ever occurrence of stroke, CHD, or hypertension during the follow-up period. Stroke was classified as either ischemic or hemorrhagic attack. The criteria for the diagnosis of CHD included interventional treatment of a coronary artery (cardiac catheterization or coronary artery bypass grafting), stable angina pectoris, unstable angina pectoris, the first occurrence of acute myocardial infarction, and congestive heart failure caused by myocardial ischemia after baseline investigation. Data on CVD events were obtained from the medical records of patients and responses obtained from the questionnaire. If a similar event occurs twice or more, the first occurrence is considered as the end event.

### Definition of MS

According to the Third Report of the Adult Treatment Unit with a Modified US National Cholesterol Education Program (2005 NCEP-ATP III)^36^, MS was defined as having three or more of the following five components: (1) central obesity: WC ≥ 90 cm in men and ≥80 cm in women; (2) TG level > 150 mg/dL (1.7 mmol/L) or has undergone corresponding treatments; (3) HDL-C level < 40 mg/dL (1.04 mmol/L) in men and < 50 mg/dL (1.30 mmol/L) in women or has undergone corresponding treatments; (4) SBP ≥ 130 mmHg and/or DBP ≥ 85 mmHg, has received appropriate treatments, or has been diagnosed with hypertension; and (5) FPG level ≥ 100 mg/dL (5.6 mmol/L), has received appropriate treatments, or was previously diagnosed with type 2 diabetes.

### FRS

The FRS standard is presented in Supplemental Table S1. FRS is used to predict the risk of CVD^18^. It is a sex-specific chart that includes age, sex, TC level, HDL-C level, smoking status, and SBP. The absolute percentage of CVD risk over 10 years was categorized as low risk (≤10%), intermediate risk (11–20%), and high risk (>20%)^18^.

### MS risk score

The MS risk score is presented in Supplemental Table S2. The five MS components were categorized based on the FRS and other guidelines in establishing an MS scoring system. The SBP, DBP, and HDL-C level were classified based on the FRS^37^. TG level was categorized according to the 2005 NCEP-ATP III^36^. FPG level was classified based on the 2005 NCEP-ATPIII criteria and the Guidelines for the Prevention, Management and Care of Diabetes Mellitus as proposed by the WHO in 2006^36^. Thus, FPG level was classified as follows: < 100, 100–110, 110–126, and ≥126 mg/dL. The WC classification criteria increased every 5 cm based on the Asian American standards. The total score was equal to the sum of the values of each MS component, and the risk of CVD was predicted based on the total score.

### Confounding Factors

The following potential confounders were used in the data analysis: sex, drinking status (current drinking, ever drinking, and never drinking), and family history of hypertension, diabetes, and CVD.

### Statistical analysis

The EpiData3.02 software was used to establish a database, and a double entry method and logic error detection were also utilized. Categorical variables were expressed as percentages, whereas continuous variables were presented as mean ± standard deviation. Chi-square test and student’s *t*-test were used to compare the baseline characteristics (percentages and means) between the groups. After adjusting for sex, drinking status, and family history of hypertension, diabetes, and CVD, the relationship between the components of MS and CVD was analyzed using the multivariate Cox proportional hazards model. The participants were divided into five groups according to the number of metabolic abnormalities at baseline: 0, 1, 2, 3, and ≥4. The participants were also classified into five groups according to quintiles of FRS; the MS risk score that included age and the MS risk score were divided into five equal parts using the same weight, and the area under the ROC of the number of MS components, MS risk score, and FRS were compared to determine their predictive ability. Based on the ROC curve analysis, the Cox proportional hazards model was used to compare the number of MS components, MS risk score, and FRS to predict the development of CVD. Meanwhile, the FRS was classified as ≤10%, 11–20%, and > 20%; the MS risk score included age, and it was then divided into three equal parts using the same weight. Under the same exposure conditions, the Cox proportional hazards model was used to compare the number of MS components and the MS risk score with the FRS as predictors of CVD. The AUC was compared using the Z test, and all statistical analyses were performed using the Statistical Package for the Social Sciences software version 17.0 for Windows (SPSS Inc., Chicago, IL, the USA). All statistical tests were two-sided, and a P-value < 0.05 was considered statistically significant. The conclusions of the manuscript were based on relevant datasets available in the manuscript.

## Electronic supplementary material

Supplemental Material
