# Supplementary material for: Comparison Between Metabolic Syndrome and the Framingham Risk Score as Predictors of Cardiovascular Diseases Among Kazakhs in Xinjiang
Source: Sci Rep. 2018 Nov 7;8:16474. doi: 10.1038/s41598-018-34587-1 (PMC6220288; doi:10.1038/s41598-018-34587-1)
Supplement: Supplementary file 1 — Supplemental Material [file 41598_2018_34587_MOESM1_ESM.pdf]

Comparison between Metabolic Syndrome and Framingham Risk Score as Predictor of Cardiovascular Diseases among Kazakhs in Xinjiang

Wenwen Yang †, Rulin Ma †, Xianghui Zhang, Heng Guo, Jia He, Lei Mao, Lati Mu, Yunhua Hu, Yizhong Yan, Jiaming Liu , Jiaolong Ma, Shugang Li, Yusong Ding, Mei Zhang, Jingyu Zhang and Shuxia Guo*

* Correspondence: gsxshzu@sina.com; Tel.: +86-180-0993-2625; Fax: +86-993-2057-153

† These authors contributed equally to this work.

SUPPLEMENTAL MATERIAL

Calculation of Framingham risk score: The risk score was calculated for each subject using the risk score of Wilson et al[1]. In this algorithm, subjects receive a point score based on categorical values of age, total cholesterol, high-density lipoprotein cholesterol, blood pressure, and smoking. The scoring sheet is available in the study by Wilson et al[1]. We calculated the risk score of each subjects.

Supplemental Table S1: Framingham Risk Score

**Framingham risk score for male**		**Framingham risk score for female**	
age	score	age	score
20-34	-9	20-34	-7
35-39	-4	35-39	-3
40-44	0	40-44	0
45-49	3	45-49	3

50-54	6
55-59	8
60-64	10
65-69	11
70-74	12
75-79	13

score					
age range					
TC	20-39	40-49	50-59	60-69	70-79
<160	0	0	0	0	0
160-199	4	3	2	1	0
200-239	7	5	3	1	0
240-279	9	6	4	2	1
≥280	11	8	5	3	1

score					
age range					
	20-39	40-49	50-59	60-69	70-79
nonsmoker	0	0	0	0	0
smoker	8	5	3	1	1

HDL-C(mg/dL)	score

50-54	6
55-59	8
60-64	10
65-69	12
70-74	14
75-79	16

score					
age range					
TC	20-39	40-49	50-59	60-69	70-79
<160	0	0	0	0	0
160-199	4	3	2	1	1
200-239	8	6	4	2	1
240-279	11	8	5	3	2
≥280	13	10	7	4	2

score					
age range					
	20-39	40-49	50-59	60-69	70-79
nonsmoker	0	0	0	0	0
smoker	9	7	4	2	1

HDL-C(mg/dL)	score

≥60	-1	≥60	-1
50-59	0	50-59	0
40-49	1	40-49	1
<40	2	<40	2

SBP(mm Hg)	untreated	treated	SBP(mm Hg)	untreated	treated
<120	0	0	<120	0	0
120-129	0	1	120-129	1	3
130-139	1	2	130-139	2	4
140-159	1	2	140-159	3	5
≥160	2	3	≥160	4	6

total points	FRS 10-year risk	total points	FRS 10-year risk
<0	<1	<9	<1
0	1	9	1
1	1	10	1
2	1	11	1
3	1	12	1
4	1	13	2
5	2	14	2
6	2	15	3

7	3
8	4
9	5
10	6
11	8
12	10
13	12
14	16
15	20
16	25
>17	>30

16	4
17	5
18	6
19	8
20	11
21	14
22	17
23	22
24	27
>25	>30

Calculation of Metabolic Syndrome(MS) risk score: Referring to the FRS[2] and other established guidelines[3,4], we assigned a value to each component of MS to establish MS scoring system.

Supplemental Table S2: Metabolic Syndrome Risk Score

Metabolic syndrome risk score for male

age	score
20～	-9
35～	-4
40～	0
45～	3
50～	6

WC(cm)	score				
	20～	40～	50～	60～	≥70
＜90	0	0	0	0	0
90～	4	3	2	1	0
95～	7	5	3	1	0
100～	9	6	4	2	1

SBP(mmHg)	untreat	treat
＜120	0	0
120～129	0	1
130～139	1	2
140～159	1	2
≥160	2	3

DBP(mmHg)	untreat	treat
＜80	0	0
80～84	0	1
85～89	1	2
90～99	1	2
≥100	2	3

55～	8
60～	10
65～	11
70～	12
＞75	13

≥105	11	8	5	3	1

HDL-C(mg/dl)	score
≥60	-1
50～59	0
40～49	1
＜40	2

TG(mg/dl)	score
<150	-1
150～199	0
200～499	1
≥500	2

FPG(mg/dl)	score
<100	-1
100～109	0
110～125	1
≥126	2

Metabolic syndrome risk score for female

age	score
20～	-7

WC(cm)	score				
	20	40～	50	60～	≥70

SBP(mmHg)	untreat	treat
＜120	0	0

DBP(mmHg)	untreat	treat
＜80	0	0

35～	-3
40～	0
45～	3
50～	6
55～	8
60～	10
65～	12
70～	14
＞75	16

	～		～		
＜80	0	0	0	0	0
80～	4	3	2	1	1
85～	8	6	4	2	1
90～	11	8	5	3	2
≥95	13	10	7	4	2

HDL-C(mg/dl)	score
≥60	-1
50～59	0
40～49	1
＜40	2

120～129	0	3
130～139	2	4
140～159	3	5
≥160	4	6

80～84	1	3
85～89	2	4
90～99	3	5
≥100	4	6

TG(mg/dl)	score
<150	-1
150～199	0
200～499	1
≥500	2

FPG(mg/dl)	score
<100	-1
100～109	0
110～125	1
≥126	2

Supplemental Table S3: The result of adjusting the MS and FRS components

items	AUC	Sensitivity（%）	*P*
MS score	0.65(0.61,0.68)	0.78	＜0.001
+TC	0.65(0.62,0.68)	0.75	＜0.001
+Smoking	0.64(0.61,0.67)	0.76	＜0.001
+Age	0.76(0.73,0.78)	0.91	＜0.001
-WC	0.64(0.61,0.68)	0.73	＜0.001
-TG	0.65(0.61,0.68)	0.78	＜0.001
-FPG	0.65(0.61,0.68)	0.77	＜0.001
FRS	0.73(0.70,0.76)	0.84	＜0.001
-TC	0.73(0.70,0.76)	0.85	＜0.001
-Smoking	0.75(0.72,0.78)	0.86	＜0.001
-Age	0.58(0.55,0.62)	0.67	＜0.001
+WC	0.74(0.72,0.77)	0.88	＜0.001
+TG	0.74(0.71,0.77)	0.87	＜0.001
+FPG	0.73(0.70,0.76)	0.84	＜0.001

Note: Specificity remained at 46.8%[2].

Supplemental Table S4. AUC of MS and its components for predicting the development of CVD

Characteristics	AUC	95%CI	*P*
WC	0.60	(0.56,0.63)	<0.001
TG	0.56	(0.52,0.59)	0.002

SBP	0.62	(0.59,0.66)	<0.001
DBP	0.64	(0.60,0.68)	<0.001
HDL-C	0.48	(0.45,0.52)	0.393
FPG	0.56	(0.53,0.60)	0.001
MS	0.59	(0.55,0.62)	<0.001

References

1 Wilson, P. W. *et al.* Prediction of coronary heart disease using risk factor categories. *Circulation* **97**, 1837-1847 (1998).

2 Yu, H., Guo, Z. R., Hu, X. S., Zhou, Z. Y. & Wu, M. [A comparison between the metabolic syndrome score and the Framingham risk score in the prediction of cardiovascular disease]. *Zhonghua Liu Xing Bing Xue Za Zhi* **31**, 208-212 (2010).

3 Executive Summary of The Third Report of The National Cholesterol Education Program (NCEP) Expert Panel on Detection, Evaluation, And Treatment of High Blood Cholesterol In Adults (Adult Treatment Panel III). *Jama* **285**, 2486-2497 (2001).

4 Grundy, S. M. *et al.* Diagnosis and management of the metabolic syndrome: an American Heart Association/National Heart, Lung, and Blood Institute Scientific Statement. *Circulation* **112**, 2735-2752, doi:10.1161/circulationaha.105.169404 (2005).
